# Diagnostic Informatics—The Role of Digital Health in Diagnostic Stewardship and the Achievement of Excellence, Safety, and Value

**DOI:** 10.3389/fdgth.2021.659652

**Published:** 2021-06-10

**Authors:** Andrew Georgiou, Julie Li, Rae-Anne Hardie, Nasir Wabe, Andrea R. Horvath, Jeffrey J. Post, Alex Eigenstetter, Robert Lindeman, Que Lam, Tony Badrick, Christopher Pearce

**Affiliations:** ^1^Australian Institute of Health Innovation, Macquarie University, Sydney, NSW, Australia; ^2^New South Wales (NSW) Health Pathology, Department of Clinical Chemistry and Endocrinology, Prince of Wales Hospital, Sydney, NSW, Australia; ^3^Department of Infectious Diseases, Prince of Wales Hospital and Community Health Services, Randwick, NSW, Australia; ^4^Prince of Wales Clinical School, University of New South Wales, Kensington, NSW, Australia; ^5^New South Wales (NSW) Health Pathology, Chatswood, NSW, Australia; ^6^Department of Pathology, Austin Health, Heidelberg, VIC, Australia; ^7^Royal College of Pathologists of Australasia Quality Assurance Programs, St Leonards, NSW, Australia; ^8^Outcome Health, Blackburn, VIC, Australia; ^9^Department of General Practice, Monash University, Clayton, VIC, Australia

**Keywords:** pathology, diagnostic error, outcomes based assessment, evaluation, safety, health informatics, decision support, test result follow-up

## Abstract

Diagnostic investigations (pathology laboratory and medical imaging) aim to: increase certainty of the presence or absence of disease by supporting the process of differential diagnosis; support clinical management; and monitor a patient's trajectory (e. g., disease progression or response to treatment). Digital health can be defined as the collection, storage, retrieval, transmission, and utilization of data, information, and knowledge to support healthcare. Digital health has become an essential component of the diagnostic process, helping to facilitate the accuracy and timeliness of information transfer and enhance the effectiveness of decision-making processes. Digital health is also important to diagnostic stewardship, which involves coordinated guidance and interventions to ensure the appropriate utilization of diagnostic tests for therapeutic decision-making. Diagnostic stewardship and informatics are thus important in efforts to establish shared decision-making. This is because they contribute to the establishment of shared information platforms (enabling patients to read, comment on, and share in decisions about their care) based on timely and meaningful communication. This paper will outline key diagnostic informatics and stewardship initiatives across three interrelated fields: (1) diagnostic error and the establishment of outcomes-based diagnostic research; (2) the safety and effectiveness of test result management and follow-up; and (3) digitally enhanced decision support systems.

## Introduction

Diagnostic investigations and tests (pathology and medical imaging) involve the observation of personal characteristics, symptoms, signs, and history. Their aim is to increase certainty of the presence or absence of disease (or in the case of the differential diagnosis process, distinguish between different possible diagnoses); support clinical management; and monitor a patient's trajectory (e.g., during or after treatment) (1). Diagnostic investigations have had a pivotal role in the response to the SARS-CoV-2 pandemic through laboratory testing and epidemiological surveillance of viral infection, not only from its *detection/diagnosis* (molecular testing by RT-PCR) but also its *prognostication, treatment*, and *monitoring* (testing related to comorbidities), and *recovery* (Anti-SARS-CoV-2 antibodies).

Digital health can be defined as the collection, storage, retrieval, transmission, and utilization of data, information, and knowledge to support healthcare (2). Digital health contributes to effective decision-making by improving the ability to gather, organize, and display information or by enhancing timely access to diagnostic reference information, facilitating follow-up, and providing feedback to clinicians and patients (3). *Diagnostic informatics* (defined as the use of digital health to facilitate the accuracy and timeliness of information transfer and enhance the effectiveness of the decision-making processes) (4) is fundamental for the safe and effective management of diagnostic (pathology and medical imaging) investigations and tests. Diagnostic informatics is a thus key component of successful *diagnostic stewardship*, which encompasses the coordinated guidance and interventions to ensure the appropriate utilization of diagnostic tests for therapeutic decision-making (5).

This paper will outline the importance of key digital health diagnostic informatics and stewardship initiatives across three interrelated areas: (1) diagnostic error and the establishment of outcomes-based diagnostic research; (2) safety and effectiveness of test result management and follow-up, including the critical role that patients can play in the process; and (3) digitally enhanced decision support systems.

## Diagnostic error and the Establishment of Outcomes-Based Diagnostic Informatics Research

Diagnostic error is a major problem in healthcare, contributing to ~10% of patient deaths and to 6–17% of hospital adverse events (6). Diagnostic error can be defined as the failure to: (1) establish an accurate and timely explanation of the patient's health problem(s) or (2) effectively communicate that explanation to the patient (6). In Australia, medico-legal data show that diagnostic error is implicated in half of medical negligence claims involving general practitioners (7).

Many factors can contribute to diagnostic error, including the lack of an integrated care pathway; the involvement of multiple specialists; problems with collaboration and communication among clinicians, patients, and their families; lack of infrastructure to support the diagnostic process; and inadequate attention to understanding the health problem and its causes (6). Existing evidence shows that in acute care an estimated 45% of laboratory testing is underutilized (i.e., when one or more tests should have been undertaken but weren't) and 21% is overutilized (i.e., tests that were unnecessary or repeated within an inappropriate time frame) (8).

There is currently a lack of a systematic outcomes-based approach to identify and monitor the prevalence and impact of diagnostic errors. In part, this is because the typical relationship between a test and a health outcome is indirect. It is made harder still by the existence of data silos across different clinical and care settings (e.g., pathology, medical imaging, medical records, emergency, and hospital administration systems), which limit the ability to link test results and referrals to the different components of the patient journey (e.g., treatment and outcome). Despite the existence of evidence-based guidelines to encourage best practice utilization of diagnostic tests, there are very few studies on how these guidelines have impacted testing patterns and patient outcomes. This was noted by a recent international scoping review that drew attention to the significant lack of strategies for optimizing test utilization and improving patient outcomes in general practice (9).

Linked and integrated digital health data sources and repositories across hospital, pathology, and primary care provide a means to identify, measure, and monitor the quality of care. They can deliver key outcome-based measures of diagnostic utilization such as patient outcome or hospital admissions, which can be used to evaluate the impact of key interventions (e.g., electronic decision support). This is the premise underlying key initiatives like the New South Wales (NSW) Health Pathology Atlas of Variation (in collaboration with the NSW Emergency Care Institute and NSW eHealth Integrated Care), designed to create a statewide quality improvement project (10).

As an example of the potential of outcome focused studies to impact on patient care, we investigated the incidence of acute kidney injury (AKI) over a 5-year period (2009–2013) across four NSW hospitals (11). Patients with AKI were identified using the serum creatinine-based definition as described in the international consensus guidelines published by the “Kidney Disease: Improving Global Outcomes” (KDIGO) Work Group. This required programming of a complex algorithm, which was run on more than 2 million creatinine results to detect the presence of AKI. The study found that only 15.9% of hospitalizations with AKI stage 1 were coded as such (11), thus highlighting the need for an evidence-based laboratory AKI decision support aid. Other examples of the value of linked data helping to power programs of research include measuring the effects of electronic medical records on healthcare (12, 13), and the impact of the rapid flu tests on emergency department (ED) performance and patient outcomes (14).

## Enhancing the Safety and Effectiveness of Test Result Management and Follow-Up

A significant source of diagnostic error can be attributed to shortcomings in the follow-up of test results, identified as a priority area by the World Health Organization's World Alliance for Patient Safety (15). International evidence shows between 41 and 100% of patients leave hospital with at least one test result pending at discharge, influenced in part by pressure on doctors to reduce length of stay (16).

Care transitions can be defined as the passage of patients between healthcare professionals, settings (units, wards, hospitals, aged care facilities), and home (17). Ineffective care transitions can lead to adverse events, such as medication errors (18), medical errors related to the completion of diagnostic workups (19), and the loss of crucial patient information (17). Arriving at a diagnosis is not a single task—it is a series of tasks and responsibilities over numerous time points, shared among many people across the healthcare spectrum (20).

Pathology and medical imaging services perform a major role in patient care by ensuring that reliable and accurate results are delivered in a timely fashion to inform clinical management. Nevertheless, there are large variations in how critically abnormal results are defined and reported, and the failure of laboratories to uniformly follow internationally recognized guidelines (21). Some important initiatives, which are seeking to address these issues, include The Royal College of Pathologists of Australasia (RCPA)—Australasian Association of Clinical Biochemists (AACB) High Risk Results Working Party, which is developing a harmonized, evidence-based “Alert List for Australasian Laboratories” (22). Similarly, the RCPA-led Pathology Information, Terminology and Units Standardizations (PITUS) program aims to establish standardized pathology information structures and terminologies to improve recording, decision support, and communication of laboratory information (23, 24).

In 2016, a national Australian stakeholder forum (involving patient groups, clinicians, managers, and professional organizations) was held in Sydney to identify patient safety challenges related to shortcomings in the governance and integration of the test result management process and the underutilization of digital health solutions. The forum drew particular attention to the role patients can contribute to effective test result management. It recommended robust follow-up procedures particularly for: (1) tests that may be pending during transitions of care; (2) critical test results; and (3) non-critical but actionable test results, which can have a severe impact on patients' health outcomes.

**Figure 1:**
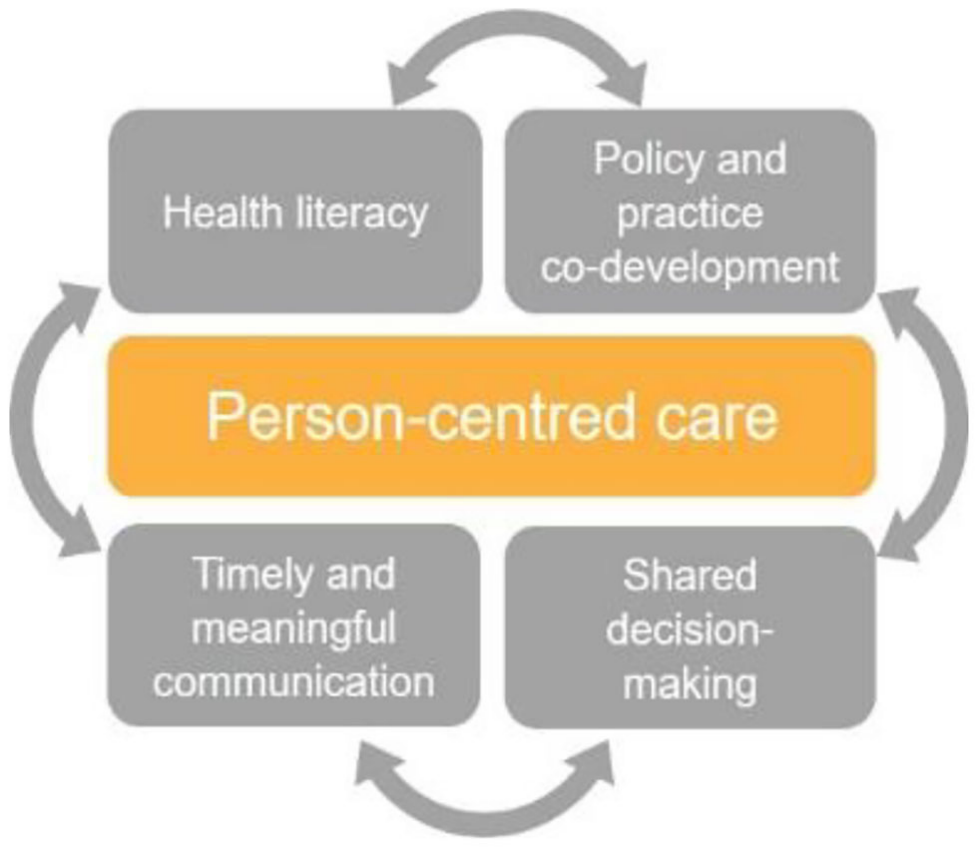
Key features of person-centered care.

Taken together, diagnostic informatics, along with diagnostic stewardship, provide a foundation stone for improved patient engagement and shared decision-making. This is because they incorporate shared information (enabling patients to read, comment on, and share in decisions about their care) and timely and meaningful communication (enabling patients to receive, send, and comprehend the information required) (25). By supporting patients to make informed decisions about their own health and the care they receive, this approach promotes person-centered care, an established key driver of quality in healthcare (26) (as depicted in Figure 1).

## Digitally Enabled Decision Support

Digital health can contribute to effective decision-making by improving the ability to gather, organize, and display information, or by enhancing timely access to diagnostic reference information, facilitating follow-up and feedback to clinicians and patients. Different types of electronic support can be used to: (1) optimize decision-making (e.g., choice of an appropriate laboratory test, improved compliance with care guidelines, improved interpretation of test results); (2) improve care processes (e.g., better documentation and improved communication); and (3) prevent errors of commission (e.g., unnecessary repetition of laboratory tests) and errors of omission (e.g., trigger immediate action in response to high-risk laboratory results) (27). Digitally enabled decision support tools can be used in a wide variety of diagnostic-related areas including as alerts and order sets (to enhance the appropriate choice of tests), the provision of important information related to decision-making (to support the identification and management of risk factors), guideline support, and the provision of relevant reference material (to promote the best available care).

The *Sensible Test Ordering Practice* (STOP) initiative was developed in Australia to promote consistent and rational diagnostic test ordering practices in acute care settings. STOP involves the use of a traffic-light system (green, amber, or red) to restrict the range of tests that can be ordered depending on the seniority of the clinicians: “green” tests may be ordered by junior doctors, while more complex, expensive, or lower-yield tests designated “amber” or “red” require senior medical staff sign off. The Australasian College for Emergency Medicine and the RCPA has developed a set of laboratory testing recommendations in EDs for common presentations based on the STOP principles (28). An example for adults presenting to EDs with chest pain is presented in Table 1. The STOP guidelines can be integrated into electronic decision support tools linked to electronic medical record systems to facilitate the consistency and appropriateness of diagnostic-test-ordering practices.

**Table 1:** STOP principles for adults presenting to ED with chest pain.

**Category**	**Recommendation**	**Test**
Green	Perform the test	Full blood count, electrolyte urea creatinine, glucose, and troponin
Amber	Consult senior medical staff	Liver function test
Red	Not generally indicated	Calcium Magnesium Phosphate, Coagulation Studies, Blood Bank, Blood gas, Urine, Microscopy, Culture & Sensitivity, Lipase, C-Reactive Protein and Creatine Kinase

While electronic decision support systems are often described as key to reducing misdiagnosis, their impact has not been strongly validated against patient outcomes, and their diffusion is patchy (29). There is also a long history of failure associated with the use of computer-based tools (29). These tools are often introduced to a clinical setting with limited pilot testing and little understanding of how they affect workflow (29). Establishing effective digital health interventions must ensure the acceptance and usability of the digital health system and include systematic feedback about their performance to users (3, 30). The STOP electronic decision support initiative will thus need to be evaluated to ensure that it is: (1) effective (e.g., helps to improve the quality of care); (2) efficient (e.g., is accessible and responsive to clinician and patient needs); (3) appropriate (e.g., promote the best possible outcomes of care); and (4) safe (e.g., help to optimize care quality and safety).

## Discussion

The diagnostic process occurs over time and often involves multiple healthcare professionals across different care settings. Arriving at a diagnosis can be an uncertain process, often reliant on limited and imperfect data or indeterminate diagnostic interpretations from pathology reports (6, 31). The measurement of diagnosis is further challenged by traditional health record structures, which can fail to capture the crucial clinician and patient narrative, communication context, and organizational culture (31). Recent advances in artificial intelligence and natural language processing tools (such as machine translation, speech recognition, and speech synthesis) provide fertile ground to improve and enhance our understanding of the diagnostic process (32).

To ensure the seamless flow of, and access to, patient information, interoperable electronic systems are critical (6). A systematic review of studies of the impact of digital health on test result management showed that, in and of itself, digital health does not provide a complete solution (33). The development of safe and effective digital health interventions needs to encompass multiple dimensions, taking into account: (1) patient-centered care (involving shared, timely, and meaningful information), (2) existing diagnostic processes (involving multiple people across different clinical settings); and (3) key organizational communication processes (multiple transactions requiring feedback, iteration, and confirmation) (33–35).

## Author Contributions

All authors contributed to the conception and design of the work, drafting of the work, revising it critically for important intellectual content, provided final approval of the version to be published, and provided agreement to be accountable for all aspects of the work in ensuring that questions related to the accuracy or integrity of any part of the work are appropriately investigated and resolved.

## Conflict of Interest

The authors declare that the research was conducted in the absence of any commercial or financial relationships that could be construed as a potential conflict of interest.
